# Toward hippocampal volume measures on ultra-high field magnetic resonance imaging: a comprehensive comparison study between deep learning and conventional approaches

**DOI:** 10.3389/fnins.2023.1238646

**Published:** 2023-12-14

**Authors:** Junyan Lyu, Perry F. Bartlett, Fatima A. Nasrallah, Xiaoying Tang

**Affiliations:** ^1^Department of Electronic and Electrical Engineering, Southern University of Science and Technology, Shenzhen, Guangdong, China; ^2^Queensland Brain Institute, The University of Queensland, St Lucia, QLD, Australia

**Keywords:** deep learning, convolutional neural network, hippocampus, longitudinal study, aging, volume estimation

## Abstract

The hippocampus is a complex brain structure that plays an important role in various cognitive aspects such as memory, intelligence, executive function, and path integration. The volume of this highly plastic structure is identified as one of the most important biomarkers of specific neuropsychiatric and neurodegenerative diseases. It has also been extensively investigated in numerous aging studies. However, recent studies on aging show that the performance of conventional approaches in measuring the hippocampal volume is still far from satisfactory, especially in terms of delivering longitudinal measures from ultra-high field magnetic resonance images (MRIs), which can visualize more boundary details. The advancement of deep learning provides an alternative solution to measuring the hippocampal volume. In this work, we comprehensively compared a deep learning pipeline based on nnU-Net with several conventional approaches including Freesurfer, FSL and DARTEL, for automatically delivering hippocampal volumes: (1) Firstly, we evaluated the segmentation accuracy and precision on a public dataset through cross-validation. Results showed that the deep learning pipeline had the lowest mean (*L* = 1.5%, *R* = 1.7%) and the lowest standard deviation (*L* = 5.2%, *R* = 6.2%) in terms of volume percentage error. (2) Secondly, sub-millimeter MRIs of a group of healthy adults with test–retest 3T and 7T sessions were used to extensively assess the test–retest reliability. Results showed that the deep learning pipeline achieved very high intraclass correlation coefficients (*L* = 0.990, *R* = 0.986 for 7T; *L* = 0.985, *R* = 0.983 for 3T) and very small volume percentage differences (*L* = 1.2%, *R* = 0.9% for 7T; *L* = 1.3%, *R* = 1.3% for 3T). (3) Thirdly, a Bayesian linear mixed effect model was constructed with respect to the hippocampal volumes of two healthy adult datasets with longitudinal 7T scans and one disease-related longitudinal dataset. It was found that the deep learning pipeline detected both the subtle and disease-related changes over time with high sensitivity as well as the mild differences across subjects. Comparison results from the aforementioned three aspects showed that the deep learning pipeline significantly outperformed the conventional approaches by large margins. Results also showed that the deep learning pipeline can better accommodate longitudinal analysis purposes.

## 1 Introduction

The hippocampus plays a vital role in memory function (Scoville and Milner, 1957), intelligence (Reuben et al., 2011), executive function, and path integration (Yamamoto et al., 2014). The hippocampal formation is highly plastic, and its atrophy has been identified as one of the earliest signs of age-related brain changes in the healthy human beings (Hasan and Glees, 1973; Hubbard and Anderson, 1981; Varma et al., 2016). Therefore, the hippocampus is of significant interest in clinical studies that aim to preserve brain volume. Evidence suggests that exercise intervention may be one of the most promising ways to increase and preserve hippocampal volume. Several studies have shown that older adults exhibit significant increases in hippocampal volume after aerobic exercise intervention (Erickson et al., 2011; Rosano et al., 2017; Teixeira et al., 2018). A recent meta-analysis study revealed a non-significant increase of 1.2% in the total hippocampal volume in the exercise group and a significant decrease of 0.72% in the control group (Wilckens et al., 2021). Therefore, highly accurate and precise approaches for measuring the hippocampal volume are essential.

Magnetic resonance images (MRIs) provide an important tool for *in-vivo* volumetric assessment of the hippocampus. Early studies investigating hippocampal volume changes following exercise adopted a manual segmentation approach (Niemann et al., 2014; Maass et al., 2015; Jonasson et al., 2016). Manual delineation is time-consuming, expertise-requiring, and of large inter- as well as intra-rater variabilities. Automatic methods based on T1-weighted MRIs have been explored and applied to measure the hippocampal volumes, with Freesurfer (Fischl, 2012) and the FMRIB Software Library (FSL) (Jenkinson et al., 2012) being two of the mostly employed segmentation tools in exercise studies (Firth et al., 2018; Wilckens et al., 2021). Voxel-based morphometry such as DARTEL is also a popular alternative to identify volumetric changes in exercise-related studies (Erickson et al., 2014; Zhou et al., 2021). These methods can well accommodate detecting the hippocampal volume change in neurodegenerative diseases such as Alzheimer’s disease (annual atrophy of 4.66%) (Barnes et al., 2009) yet show vulnerable test–retest reliabilities in exercise-related studies. Utilizing FSL, a considerable number of healthy adults exhibited annual volume changes of over 5% in the hippocampus (Erickson et al., 2011; Rosano et al., 2017), which fell outside the range of the typically-reported age-related annual hippocampal atrophy of 0.8 to 1.7% (Raz et al., 2005). Utilizing Freesurfer, the results revealed no significant group effect on the volume change in the hippocampus after exercise intervention (Jonasson et al., 2016; Thomas et al., 2016). Consequently, these studies were inconsistent in the enhancing effects of exercise on hippocampal volume. The variability raises inquiries about the extent to which or whether exercise can be considered a clinically effective approach for increasing hippocampal volume.

Ultra-high field MRI (e.g., 7 T) is gaining popularity due to its higher signal-to-noise ratios, high-resolution acquisitions and better contrast, visualizing more details in the hippocampus and its boundary (Okada et al., 2022). This provides an opportunity to obtain conclusive results in exercise-related studies. However, current automated approaches fail to leverage the high signal-to-noise ratio and submillimeter resolution of ultra-high field MRI. Only two existing exercise studies use ultra-high field imaging, one relying on manual segmentation (Maass et al., 2015) and the other confronting a large volume change issue when using FSL (Rosano et al., 2017). This is because current methods are based on 1.5T or 3T atlases and work in a millimeter space, which cannot effectively accommodate 7T images due to the high intensity inhomogeneity and data complexity (Bazin et al., 2014). In such context, there is an increasing need for an automated volumetric analysis tool that has strong test–retest reliability and can well generalize to ultra-high field MRIs for exercise studies.

Deep learning has been used for brain image segmentation (Greve et al., 2021; Pardoe et al., 2021; Svanera et al., 2021; Balboni et al., 2022), but it differs from conventional approaches in that it learns non-linear mappings at both intensity and semantic levels in an end-to-end manner with no need of utilizing hand-crafted features. Deep learning-based segmentation methods have been found to improve over conventional approaches by considerable margins in terms of both segmentation accuracy and inference efficiency (Dolz et al., 2018; Brusini et al., 2020; Wu and Tang, 2021). Nevertheless, deep learning-based hippocampal segmentation has not been adequately validated in longitudinal settings. In addition, the test–retest reliability of deep learning-based hippocampal segmentation methods, especially in healthy adults and ultra-high field MRIs, remains under investigated.

To address these gaps, we present and validate a fully-automated deep learning pipeline equipped with nnU-Net which is an extensively validated deep learning method (Isensee et al., 2021) for hippocampal segmentation from T1-weighted MRIs. To enhance robustness and avoid overfitting, we included training datasets from multiple scanners and populations. Cross-validation was performed to validate the accuracy of the evaluated deep learning pipeline in terms of volume percentage error and Pearson correlation coefficient of the bilateral hippocampal volumes. Additionally, we benchmarked the test–retest reliability on three submillimeter MRI datasets of healthy subjects and one disease-related MRI dataset from different scanners and acquisition protocols, including two 7T MRI datasets and two 3T MRI datasets, using intraclass correlation coefficient (Shrout and Fleiss, 1979), volume percentage difference and Bayesian linear mixed effect modeling (Tustison et al., 2018). Comprehensive comparisons with three conventional approaches including Freesurfer, FSL and DARTEL were conducted. Our study aims to determine whether deep learning outperforms conventional approaches for hippocampal volume measurement in healthy subjects and whether it is possible to detect subtle changes based on 7T MRIs.

## 2 Materials and methods

### 2.1 Subjects

We included three publicly available datasets with test–retest or longitudinal MRIs from healthy aging subjects to validate and compare the test–retest reliability between deep learning and conventional hippocampal segmentation approaches. The structural MRIs in these three datasets were all of submillimeter isotropic resolutions, acquired from either 3T or 7T scanners. We also included longitudinal data from the Alzheimer’s Disease Neuroimaging Initiative (ADNI)-3 to further validate the effectiveness of the deep learning pipeline.

- The Human Connectome Project (HCP) test–retest dataset (Van Essen et al., 2013) included 45 healthy adults aged 22–35 years (M = 13, *F* = 32) with structural imaging data. T1-weighted images were acquired from a customized Siemens 3 T “Connectome Skyra” scanner (TR/TI/TE = 2400/1000/2.14 ms) at the Washington University in St. Louis. The voxel dimensions were 0.7 mm × 0.7 mm × 0.7 mm, and the intervals between test and retest scans varied from 1 to 11 months.
- The Toward Optimizing MRI Characterization of Tissue (TOMCAT) imaging dataset (Shaw et al., 2020) included 7 healthy subjects (age: 26.29 ± 3.35 years, sex: *M* = 4, *F* = 3). T1-weighted images were acquired from a 7T Siemens Magnetom scanner (TR/TI/TE = 4300/840/2.5 ms) at the University of Queensland, all with a 0.75 mm isotropic voxel size. There were three scanning sessions for each subject with a three-year interval between session one and session two and a 45-min interval between session two and session three, the latter two sessions of which were used in the evaluation of test–retest reliability.
- The CEREBRUM-7T Glasgow dataset (Svanera et al., 2021) included 76 healthy subjects (age: 25.89 ± 4.89 years), 34 of which had longitudinal MRIs with between 2 and 7 follow-up sessions varying from 1 month to 6 months. T1-weighted images were acquired from a 7T Siemens Magnetom scanner (TR/TI/TE = 4680/−/2.07 ms) at the Queen Elizabeth University Hospital, UK, all with a 0.63 mm isotropic voxel size.
- The ADNI3 dataset (adni.loni.usc.edu) included 64 subjects (age: 77.28 ± 7.07 years, sex: *M* = 36, *F* = 28), consisting of 5 cognitively normal (CN), 59 mild cognitive impairment (MCI). Data was downloaded in August 2023 and the inclusion criteria was that participants had 3D isotropic full-brain T1w MRIs and been scanned three times. The scanning protocol is provided in http://adni.loni.usc.edu/wp-content/uploads/2017/07/ADNI3-MRI-protocols.pdf. The clinical data is provided in Supplementary materials.

### 2.2 Image analysis

#### 2.2.1 Deep learning pipeline

In this study, we adopted nnU-Net as our deep learning approach to segment the hippocampus. nnU-Net (Isensee et al., 2021) is a deep learning-based segmentation toolbox that automatically configures preprocessing, network parameters, training strategy and post-processing according to the imaging modality, image sizes, voxel spacings and class ratios. Without any expert knowledge and manual intervention, nnU-Net can achieve state-of-the-art performance in most biomedical segmentation tasks.

We used a coarse-to-fine pipeline to further leverage nnU-Net. We first applied an nnU-Net to coarsely segment the hippocampus from the native whole brain T1-weighted images. The local region surrounding the hippocampus was then cropped according to the coarse segmentation. A second nnU-Net was applied to finely segment the hippocampus from the cropped volumes, resulting in the final segmentation results. By ignoring the noise and redundant information outside the local volume surrounding the hippocampus, this pipeline provided more accurate segmentation. To fully exploit the spatial information at a submillimeter resolution, normalization techniques such as registration to the standard MNI152 1 mm space were not employed. Brain extraction was also not required since the pipeline worked in a coarse-to-fine manner. All input data were resampled into 0.5 × 0.5 × 0.5 mm^3^ isotropic resolution through bicubic interpolation.

We included the following three publicly available datasets differing in scanner, population, and image protocol to train the deep learning pipeline.

- The Alzheimer’s Disease Neuroimaging Initiative-Harmonized Hippocampal Protocol (ADNI-HarP) dataset (Boccardi et al., 2015) is a subset of the original ADNI dataset, containing T1-weighted MRIs of 135 individuals with hippocampal delineations from five qualified tracers. The labels were corrected and finalized according to the HarP protocol. Among all subjects, 44 were cognitively normal, 46 were mild cognitive impaired (MCI), and 45 were Alzheimer diseased. 68 scans were acquired from a 1.5 T scanner, and the other 67 scans were acquired from a 3 T scanner. Detailed information about the scanning machines and the corresponding acquisition protocols can be found at https://www.adni-info.org/scientists/MRIProtocols.aspx. All scans were rigidly aligned to the MNI ICBM 152 space with 1 × 1 × 1 mm^3^ voxel dimensions.
- The Medical Segmentation Decathlon (MSD) hippocampus dataset (Simpson et al., 2019) consisted of T1-weighted MRIs from 90 healthy subjects and 105 subjects with a non-affective psychotic disorder, taken from the Psychiatric Genotype/Phenotype Project at Vanderbilt University Medical Center. The images were acquired from a 3 T Philips Achieva scanner (TR/TI/TE = 860/800/3.7 ms). Manual tracing of the hippocampus following a specific protocol (Pruessner et al., 2000; Woolard and Heckers, 2012) was provided. Only the cropped regions of interest were provided, and the image voxel sizes were all 1 mm^3^.
- The Penn Memory Center (PMC) atlas set (Xie et al., 2019) consisted of MRIs from 15 cognitively normal controls and 14 amnestic MCI patients scanned on a 3 T Siemens Trio scanner (TR/TI/TE = 1600/950/3.87 ms) at the University of Pennsylvania. The native T1-weighted images were all of 1 × 1 × 1 mm^3^ isotropic resolution and have been denoised for Rician noise to 0.5 × 0.5 × 1.0 mm^3^. The procedure of manually segmenting the hippocampus also followed the HarP protocol.

For nnU-Net in the coarse stage, we trained the model on whole-brain volumes from the ADNI-HarP dataset. For nnU-Net in the fine stage, we used cropped volumes surrounding the hippocampus from the ADNI-HarP, MSD, and PMC datasets to train the model. Specifically, we cropped the volumes based on the provided ground-truth labels for the ADNI-HarP dataset, while the cropped volumes were already provided for the MSD and PMC datasets. We split the datasets into training and validation sets, with an 80:20 ratio. The validation sets were exclusively used for monitoring the training process and preventing overfitting, and were never used during training. The final model was chosen based on the best segmentation performance on the validation set. The training and validation sets consisted of images from different datasets with equal proportions.

The pipeline was implemented using Python version 3.9.5 with PyTorch version 1.11.0 and nnU-Net version 1.7.0. The whole pipeline took approximately 6 h to train on a workstation equipped with an Intel Xeon Gold CPU 6132 and NVIDIA Tesla V100, with 4 h for the coarse stage and 2 h for the fine stage. The inference time for each scan depended on the image size, typically being less than 10 s/scan.

#### 2.2.2 Freesurfer pipeline

Registration-based hippocampal segmentation was carried out using Freesurfer v7.2 (Massachusetts General Hospital, Harvard Medical School^1^). Briefly, T1-weighted images were affine registered to the MNI305 space and brain-extracted, followed by an initial volumetric labeling process. The subcortical volume segmentation was finalized through a high dimensional nonlinear volumetric alignment to the MNI305 atlas. Detailed technical information can be found elsewhere (Fischl et al., 2002).

#### 2.2.3 FSL pipeline

Hippocampal segmentation was also performed using a model-based approach with FSL’s FMRIB Integrated Registration and Segmentation Tool (FIRST). The shape and appearance models based on multivariate Gaussian assumptions used in FIRST were constructed from manual segmentation, as detailed in Patenaude et al. (2011). During inference, FIRST searches through linear combinations of shape models of variation to obtain the most probable shape instance based on the intensity information of a given T1-weighted image of interest.

#### 2.2.4 DARTEL pipeline

We also utilized a voxel-based morphometry approach for longitudinal structural brain imaging analysis (Mills and Tamnes, 2014), the method of which is also commonly seen in exercise studies concerning hippocampal volume measurements. Such voxel-based morphometry analysis was conducted using DARTEL (Ashburner, 2007), a component of the Statistical Parametric Mapping software package (SPM12)^2^ that runs on MATLAB R2021a. DARTEL initially segmented a T1-weighted image into grey matter (GM), white matter (WM), and cerebrospinal fluid (CSF). Subsequently, the segmented GM and WM volumes were rigidly aligned across all subjects to create an initial template. The images were then iteratively warped to the template using diffeomorphic registrations. After warping, the images were affine transformed to MNI152 space, modulated with Jacobian determinant and smoothed using a Gaussian kernel. We empirically set the full-width at half maximum (FWHM) of the Gaussian smoothing kernel to be 4, with lower values having been suggested for subcortical regions with less variability (Shen and Sterr, 2013). All the other parameters in DARTEL were set to be their default values. The resulting smoothed images represented the regional tissue volumes, with the hippocampal volume obtained by masking the hippocampal region with MNI152.

### 2.3 Statistical analysis

We compared the performance of different volume measurement approaches from multiple perspectives, including accuracy, test–retest reliability, and longitudinal modeling.

The hippocampal volume accuracy was measured by the volume percentage error (VPE):


$$\mathrm{VPE}=100\cdot \frac{v_{\mathrm{pred}}-v_{\mathrm{gt}}}{v_{\mathrm{gt}}}$$

where $v_{pred}$ is the predicted volume and $v_{gt}$ is the ground-truth volume from manual segmentation. A VPE closer to zero indicates a lower systematic error, and a lower standard deviation suggests a higher precision. However, the hippocampal delineation protocols for the atlases used in different methods may differ. We further employed the Pearson correlation coefficient (CC) between the predicted and ground-truth volumes, wherein CC > 0.9 indicates very strong correlation, 0.7 < CC < 0.9 indicates strong correlation, 0.4 < CC < 0.7 indicates moderate correlation and CC < 0.4 indicates weak or negligible correlation (Schober et al., 2018).

To assess the test–retest reliability of the volume measurements, we first used the intraclass correlation coefficient (ICC) (Shrout and Fleiss, 1979; Bland and Altman, 1996) based on a single rater (*k* = 1), consistency, two-way mixed-effects model. We interpreted ICC values less than 0.75 as poor reliability, values between 0.75 and 0.95 as good reliability, and values greater than 0.95 as excellent reliability (Koo and Li, 2016). Second, we calculated the volume percentage difference (VPD):


$$VPD=100\cdot \frac{2*\left|v_{test}-v_{retest}\right|}{v_{test}+v_{retest}}$$

where $v_{test}$ points to the volume measure of the test session and $v_{retest}$ points to the volume measure of the re-test session. A smaller value indicates a better reliability.

For data with more than two sessions, we adopted a longitudinal Bayesian linear mixed effects (BLME) model (Tustison et al., 2018) to assess the relationships between longitudinal and cross-sectional results while accounting for subject-specific trends. For each region of interest, the BLME model can be formulated as


$$V_{ij}\sim N\left(\alpha_{i}+\beta_{i}t,\sigma^{2}\right)$$

$$\alpha_{i}\sim N\left(\alpha_{0},\tau^{2}\right)\;\beta_{i}\sim N\left(\beta_{0},\rho^{2}\right)$$

$$\alpha_{0},\beta_{0}\sim N\left(0,10\right)\;\sigma ,\tau ,\rho \sim Cauchy^{+}\left(0,5\right)$$

where $V_{ij}$ denotes the $i^{th}$ subject’s volume measurement at the $j^{th}$ timepoint and t denotes the normalized time from baseline by month. In a BLME model, τ is interpreted as the between-subject variability and σ is interpreted as the within-subject variability. A larger τ and a smaller σ are simultaneously demanded, since we expect the volume as a biomarker to discriminate between subjects but still preserve within-subject reproducibility. As such, we finally employed a summary measure named as the variance ratio


$$r=\frac{\tau }{\sigma }$$

wherein a higher value indicates a less biased volume measurement.

To assess the statistical significance of our results, we first verified whether the distributions of VPE and VPD were Gaussian using Kolmogorov–Smirnov normality tests ( p<0.05 ). We then used paired t-tests or Wilcoxon matched-pairs signed rank tests, depending on whether the distribution is Gaussian or non-Gaussian, to test for significant differences between two distributions. Additionally, we performed an F-test to compare the variances of VPE values and determined whether the deep learning pipeline was more precise than conventional methods. We performed a Fisher’s *z*-test to compare the CCs whether the deep learning pipeline was more accurate than conventional methods. We defined statistical significance for these tests as p<0.01 . For ICC scores, we considered two scores to be significantly different if there was no overlap between the confidence interval (CI) of one ICC score and the point estimate of the other. For the variance ratio, between-subject variability, and within-subject variability, we applied the same logic to determine statistical significance as that for ICC scores. More statistical details refer to Supplementary materials.

## 3 Results

### 3.1 Accuracy and precision

We compared the accuracy and precision of the hippocampal volume measurement on the ADNI-HarP dataset for the four different methods of interest: the deep learning pipeline based on nnU-Net (DL), Freesurfer, DARTEL, and FSL. To obtain the results for DL, we conducted 5-fold cross-validation. As shown in Table 1, DL exhibited the lowest systematic error, with mean VPE values of 1.3 and 1.5% for the left and right hippocampus. The Wilcoxon matched-pairs signed rank test revealed that DL had significantly lower absolute VPE values than Freesurfer, DARTEL, and FSL in the bilateral hippocampal volumes. Furthermore, DL exhibited the best precision, with standard deviations of 5.2 and 6.2% for the left and right hippocampus, as determined by the *F*-test.

**Table 1:** Statistical values including median, mean, 25th percentile, 75th percentile and standard deviation of volume percentage error (VPE) for each method for hippocampal volume measurements on ADNI-HarP.

	Median		Mean		25th percentile		75th percentile		Standard deviation	
	*L*	*R*	*L*	*R*	*L*	*R*	*L*	*R*	*L*	*R*
DL	1.3%	1.3%	1.5%	1.7%	−1.5%	−2.0%	3.8%	4.7%	5.2%	6.2%
Freesurfer	24.7%	21.9%	26.2%	23.4%	16.3%	16.4%	34.0%	30.8%	13.7%	10.7%
DARTEL	−3.8%	−4.5%	−3.3%	−4.4%	−14.5%	−14.6%	6.2%	3.7%	16.1%	16.7%
FSL	22.1%	21.4%	20.9%	21.3%	13.1%	11.9%	29.7%	31.2%	12.5%	14.5%

A VPE closer to zero indicates a lower systematic error, and a lower standard deviation suggests a higher precision.

**Figure 1:**
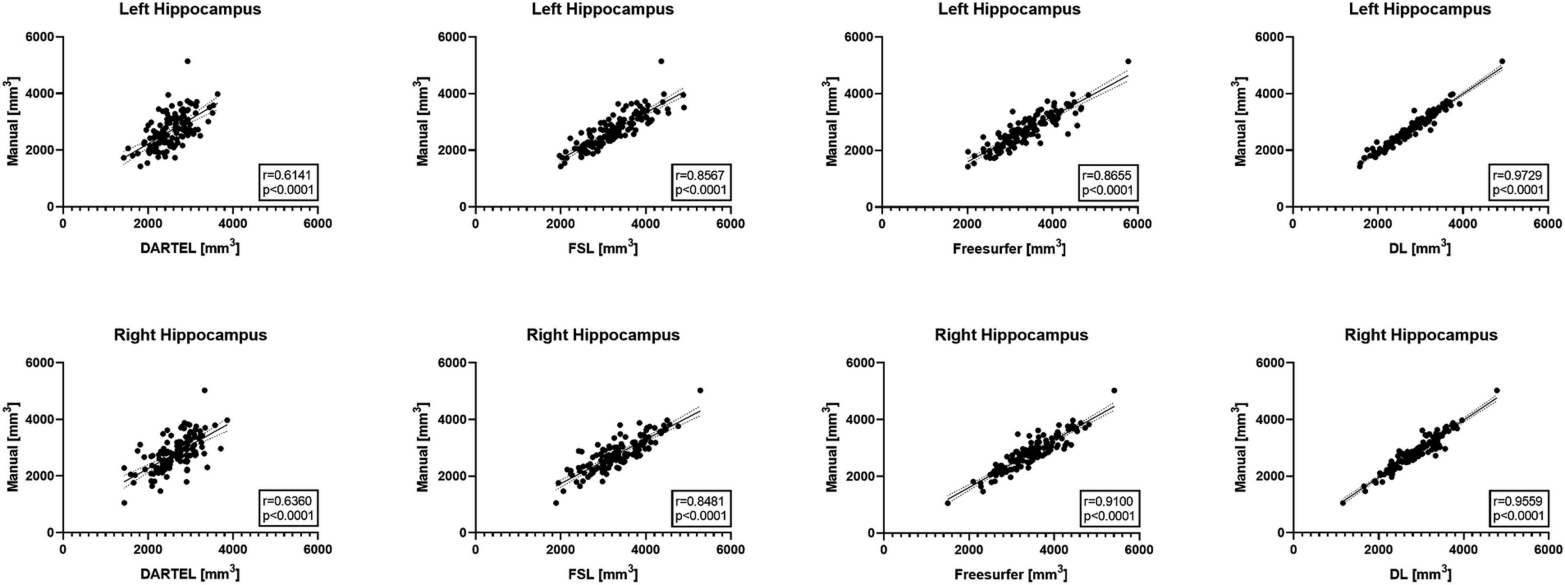
Pearson correlation coefficient analyses between automated methods and manual delineation on ADNI-HarP: scatter plots wherein each solid line shows a linear fit with 95% confidence interval. A higher *r* value indicates stronger correlation.

Figure 1 illustrates the correlations between the automatically-measured hippocampal volumes obtained by DL, Freesurfer, DARTEL, as well as FSL and the manually-derived volumes. The automatic volumes obtained from all the four methods showed significant positive correlations with the manually-derived ones. DL significantly outperformed the other methods, with CCs of 0.9729 and 0.9559 for the left and right hippocampus, respectively, indicating very strong correlations with the manually-derived volumes. Our results demonstrated outstanding accuracy and precision of DL for hippocampal volume measurement.

### 3.2 Test–retest reliability

We compared the test–retest reliability on the HCP and TOMCAT datasets. Table 2 summarizes the ICC scores of the hippocampal volumes measured by DL, Freesurfer, FSL, and DARTEL. On the HCP dataset, DL exhibited significantly higher ICC scores for both left and right hippocampus than both Freesurfer and FSL but lower than those obtained from DARTEL. On the other hand, DL yielded the highest ICC scores among all the four methods on the TOMCAT dataset.

**Table 2:** Intraclass correlation coefficients (ICCs) with their 95% confidence intervals for hippocampal volumes from different methods on HCP and TOMCAT.

	HCP		TOMCAT	
	*L*	*R*	*L*	*R*
DL	0.985 (95% CI: [0.973, 0.992])	0.983 (95% CI: [0.970, 0.991])	0.990 (95% CI: [0.944, 0.998])	0.986 (95% CI: [0.919, 0.998])
Freesurfer	0.893 (95% CI: [0.813, 0.939])	0.923 (95% CI: [0.864, 0.957])	0.959 (95% CI: [0.781, 0.993])	0.969 (95% CI: [0.830, 0.995])
DARTEL	0.995 (95% CI: [0.991, 0.997])	0.995 (95% CI: [0.991, 0.997])	0.976 (95% CI: [0.869, 0.996])	0.959 (95% CI: [0.783, 0.993])
FSL	0.935 (95% CI: [0.885, 0.964])	0.922 (95% CI: [0.862, 0.956])	0.816 (95% CI: [0.257, 0.966])	0.047 (95% CI: [−0.682, 0.729])

A higher ICC value indicates better test–retest reliability.

**Figure 2:**
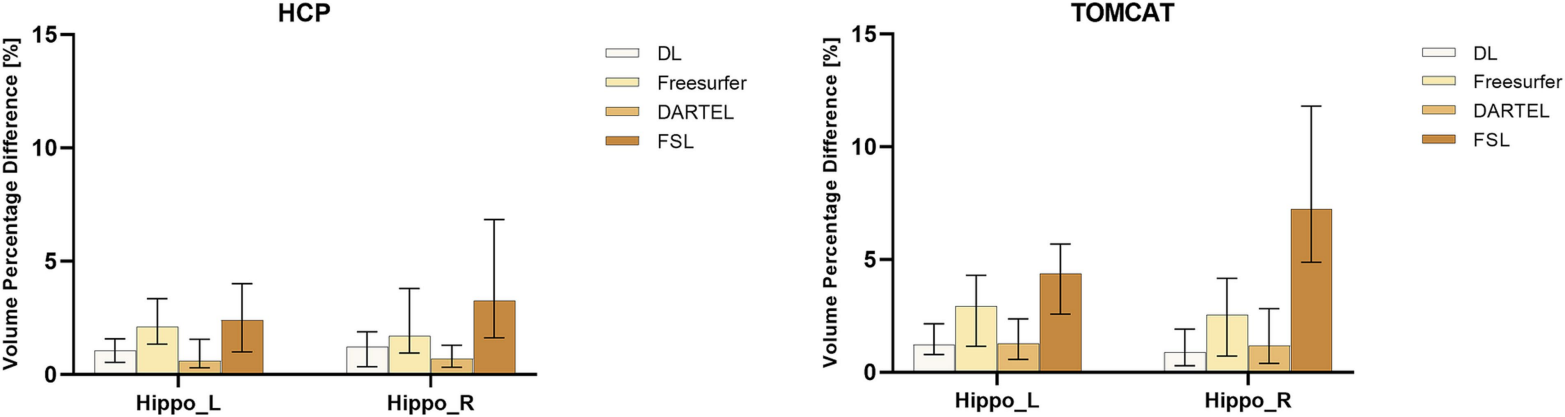
Boxplots with medians and interquartile ranges of volume percentage differences (VPDs) for different methods on HCP and TOMCAT. A lower VPD indicates better test–retest reliability.

Figure 2 presents VPD’s medians and interquartile ranges between test and re-test sessions for the four different volume measurement approaches. DL demonstrated the second-highest consistency in volume on the HCP dataset and the highest consistency on the TOMCAT dataset. The Wilcoxon matched-pairs signed rank test showed that DL had significantly lower VPD than Freesurfer and FSL for both left and right hippocampus on the HCP dataset. However, it achieved higher VPD than DARTEL on the right hippocampal volume. On the TOMCAT dataset, we observed that DL yielded significantly lower VPD than Freesurfer and FSL in the left hippocampal volume.

### 3.3 Longitudinal Bayesian linear mixed effects modeling

**Figure 3:**
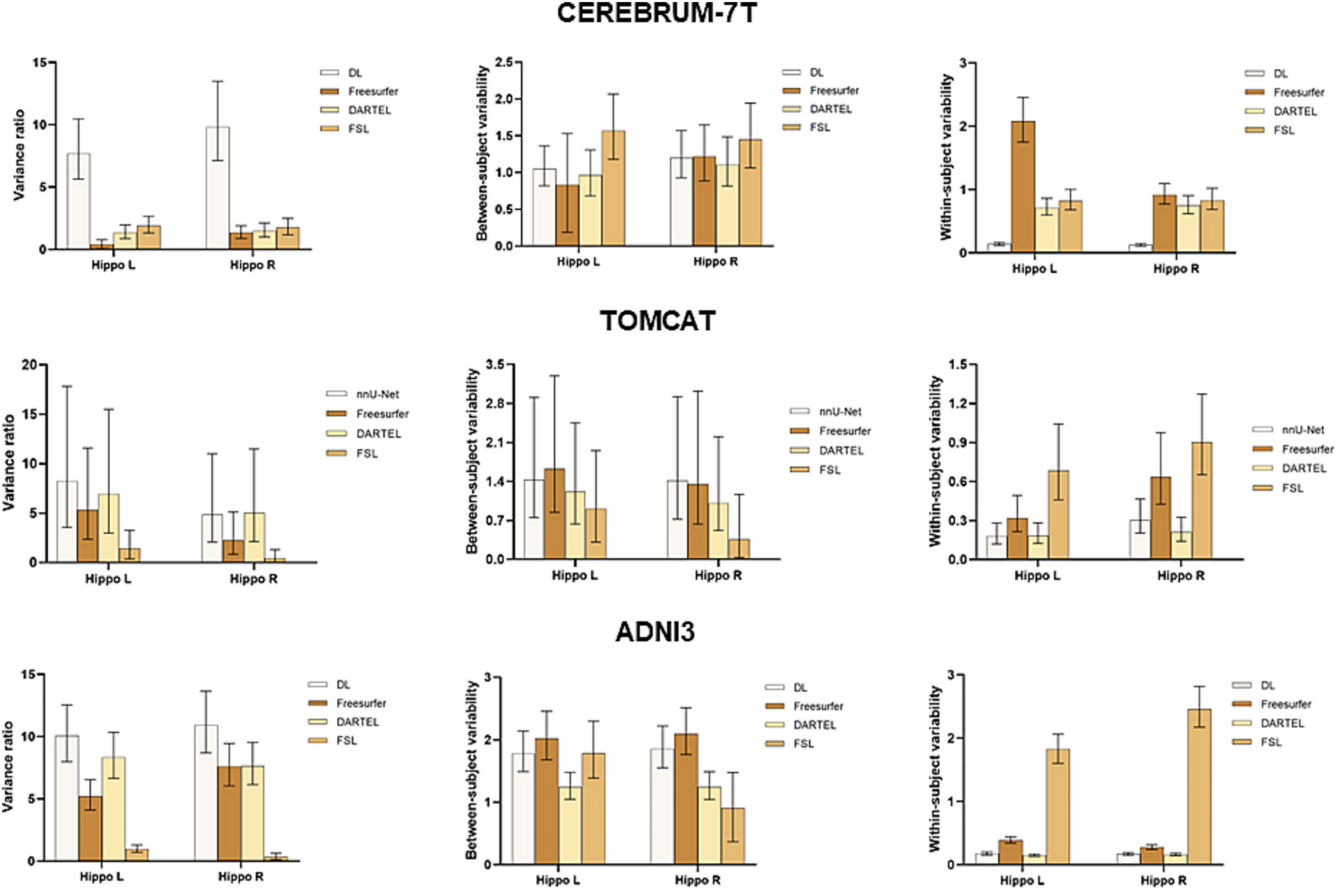
Boxplots with means and 95% confidence intervals of the variance ratio, between-subject variability and within-subject variability for all four methods under comparison on CEREBRUM-7T, TOMCAT, and ADNI3. The higher variance ratios indicate better discrimination between subjects, and higher within-subject reproducibility between the test–retest conditions.

To further assess the performance of the four volume measurement approaches, we compared the variance ratio, between-subject variability, and within-subject variability using a BLME model on the CEREBRUM dataset. The means and 95% confidence intervals (CIs) of the measures are presented in the upper row of Figure 3. The results showed that DL had the highest variance ratio, the second highest between-subject variability, and the lowest within-subject variability among all the four methods under comparison. Specifically, DL exhibited significantly higher variance ratio than all other methods for both left and right hippocampus. For between-subject variability, DL had a higher value than DARTEL for both left and right hippocampus, and Freesurfer for the left hippocampus only, with overlapping 95% CIs. However, it showed lower values than FSL. Regarding within-subject variability, DL achieved significantly lower values than all other methods for both left and right hippocampus. The middle row of Figure 3 presents the results on the TOMCAT dataset. DL demonstrated the highest variance ratio, the second highest between-subject variability and the lowest within-subject variability for the left hippocampus and the second highest variance ratio, the highest between-subject variability and the second lowest within-subject variability for the right hippocampus, yet not significant. These findings demonstrated the superior performance of DL, in terms of variance ratio and within-subject variability, indicating that it can capture subtle changes in the hippocampal volume measurements.

The lower row of Figure 3 illustrates the results on the ADNI3 dataset. DL obtained the highest variance ratios for both left and right hippocampus. Specifically, DL had a significantly higher between-subject variability than the second-best method DARTEL, while having comparable within-subject variability. These findings indicated that DL not only excels in the context of healthy, younger individuals, but also displays robust reliability when it comes to detecting disease-related changes within datasets marked by extended follow-up periods and subjects afflicted by medical conditions.

## 4 Discussion

In this study, we evaluated the performance of a deep learning pipeline based on nnU-Net and three conventional approaches, including Freesurfer, DARTEL, and FSL, for measuring hippocampal volumes from submillimeter T1-weighted MRIs. The results showed deep learning could enhance hippocampal volume measurement in terms of accuracy, precision, and test–retest reliability, particularly for ultra-high field MRIs, and is able to detect subtle changes in the hippocampal volumes of healthy adults after an exercise intervention.

Cross-validation experiments on ADNI-HarP were conducted to validate the accuracy and precision of DL. The results demonstrated that DL significantly reduced the systematic difference in volume measurements compared to DARTEL, namely −3.3 to 1.5% for the left hippocampus and − 4.4 to 1.7% for the right hippocampus, in terms of the mean VPE values. Such improvements were consistent with those reported by a previous study (Balboni et al., 2022) and shall be attributed to the ability of deep learning to utilize both interpretable features (e.g., shape, intensity) and latent features in high-dimensional spaces. Furthermore, the convolutional layers used in nnU-Net allowed the model to capture long-term voxel dependencies, leading to superior performance. On the other hand, segmentation-based conventional methods, such as Freesurfer and FSL, achieved substandard performance due to their different boundary definitions of the hippocampus (Fischl et al., 2002; Patenaude et al., 2011). This conclusion was also supported by their 25^th^ and 75^th^ percentiles. VPE’s standard deviation indicated precision, with DL significantly outperforming Freesurfer by 8.5 and 4.5% for the left and right hippocampus, and similar degrees over other conventional methods. We also used the correlation coefficient to evaluate the correlation between automated volume measurements and manual ones, excluding the effect of different hippocampal definitions. The results showed a very strong correlation for DL and strong correlations for Freesurfer and FSL. However, DARTEL only showed moderate correlation since it smoothed voxels of sharper changes and tended to predict a conservative volume close to the one in the atlas. This is illustrated in Figure 1, where DARTEL seldom predicted extreme volumes. It should be noted that the conventional methods were not trained or developed on the same set of manual annotations while DL was trained on those datasets. This is also one of the factors contributing to their significant differences.

We did not compare the accuracy and precision between the methods on MSD since it only provides cropped regions of interest, whereas Freesurfer, FSL, and DARTEL operate exclusively on complete MRIs. Similarly, PMC’s limitation lies in its provision of denoised images, which significantly impacts the efficacy of Freesurfer and DARTEL, as these methods typically demand original or minimally processed images. To ensure fair comparison, we only evaluate the accuracy and precision of these methods on ADNI-HarP. Nevertheless, we assessed the segmentation performance of DL on MSD and PMC. Supplementary Tables S1, S2 show the VPE statistics on MSD and PMC, respectively. Supplementary Figure S1 illustrates the Pearson correlation coefficients (CCs) between DL-derived hippocampal volumes and manually obtained volumes on HarP, MSD, and PMC datasets. We also investigate whether nnU-Net exhibits segmentation bias across these three manually segmented datasets. Fisher’s *z*-test revealed that CC on PMC is significantly larger than those on HarP and MSD, and CC on HarP is significantly larger than that on MSD. This discrepancy can be attributed to the congruence between HarP and PMC in manual tracing protocol, with the latter having superior image quality due to prolonged imaging acquisition. Deep learning model will fit better to those cases with distinct boundaries and superior contrast, aligning with PMC’s attributes. Hence, DL achieved notably higher accuracy on PMC. These outcomes underscore the prospect of DL capitalizing on ultra-high field MRIs more effectively.

We qualitatively compared the segmentation results from DL, Freesurfer and FSL on the TOMCAT and HCP datasets. DARTEL was not included since it did not estimate the volume by directly segmenting the hippocampus. As shown in Supplementary Figure S2, DL’s segmentation outcomes exhibited superior qualities, characterized by smoother and more congruent boundaries aligned adeptly with the hippocampal regions, when compared to Freesurfer and FSL. Importantly, both Freesurfer and FSL exhibited a tendency toward over-segmentation, particularly apparent within the CA1 and SUB regions. The comparisons illustrated the superiority of DL. Moreover, the boundary artifacts evident in the segmentations from Freesurfer and FSL revealed their limited ability to harness high-resolution information effectively.

Test–retest reliability was assessed by ICC and VPD. We found excellent levels of consistency for DL on the HCP and TOMCAT datasets. The ICC values on TOMCAT (7T) were even higher than those on HCP (3T), indicating that DL took advantages of higher signal-to-noise ratio and better contrast provided by ultra-high field MRIs. In contrast, DARTEL and FSL suffered from high intensity inhomogeneity, inducing decreased performance to varying degrees. DARTEL’s parametric bias correction model developed to deal with intensity nonuniformity was not suitable for ultra-high field MRI, leading to decrease in performance (Ashburner and Friston, 2005). FSL’s intensity priors for the appearance models only worked for 1.5T and 3T MRIs, resulting in poor reliability on TOMCAT and even failure in some cases. Freesurfer performed slightly better in terms of ICC on TOMCAT but not HCP, which could be due to the relatively small sample size (*N* = 7) of TOMCAT. In terms of VPD, DL had medians of 1.04 and 1.21% on HCP and 1.23 and 0.88% on TOMCAT, respectively for the left and right hippocampus. Specifically, 81.1% of the VPD values on HCP and 78.6% on TOMCAT were less than 2%. These test–retest reliability values can be useful for assessing hippocampal volume changes after exercise intervention (1.2%) (Wilckens et al., 2021) and annual age-related atrophy rate (0.8–1.7%) (Raz et al., 2005). We also found that DARTEL outperformed FSL and Freesurfer in terms of VPD. However, DARTEL was less commonly used in previous exercise studies, possibly due to its inability to allow for manual inspection or correction, and the regularization effect of Gaussian smoothing may weaken its ability to detect subtle changes. These limitations might also be the reason why DARTEL achieved significantly lower VPD values than Freesurfer. To investigate the effect, we employed distinct Full Width at Half Maximum (FWHM) parameters on TOMCAT. We specifically tested FWHM values at 1, 2, and 4 mm. As presented in Supplementary Figure S3, higher FWHM settings corresponded to lower mean volume percentage difference (VPD) values. This trend suggested that selecting smaller FWHM values may potentially mitigate the extent of smoothing implemented by the DARTEL procedure. This, in turn, could augment the method’s responsiveness to subtle voxel variations, encompassing both expansion and contraction phenomena. Despite this observation pointing toward higher sensitivity with smaller FWHM settings, our analyses did not yield any statistically significant difference due to the sample size.

BLME was used to fit the longitudinal data from the CEREBRUM dataset. Between-subject variability, within-subject variability, and variance ratio were used to evaluate the model. A higher variance ratio characterized by both higher between-subject variability and lower within-subject variability indicates better performance in a longitudinal study. Our results showed that DL outperformed other methods, with a significantly higher variance ratio. Specifically, it had means of 7.70 and 9.83 for the left and right hippocampus, while the second-best method, FSL, had means of 1.92 and 1.77. The advantage of DL was established by its superior within-subject variability, which serves as an indirect measure of test–retest reliability. Additionally, it demonstrated adequate between-subject variability, suggesting that it did not simply predict uniform volumes to achieve high test–retest reliability. In contrast, Freesurfer and DARTEL showed significantly decreased test–retest reliability compared to that on ADNI3, indicating their vulnerability to ultra-high field MRIs. DARTEL relies heavily on the quality of tissue segmentation (GM, WM, CSF), attaining unsatisfactory results on the CEREBRUM dataset. Freesurfer, on the other hand, may experience abnormal cross-correlation between CEREBRUM 7 T images and its 1.5 T MNI305 atlas, leading to failure of convergence in registration and unsatisfactory hippocampal segmentation. Although Freesurfer v7 has been improved for ultra-high field MRIs, similar findings were also reported in previous studies (Svanera et al., 2021). The diminished performance of Freesurfer on 7 T data underscored the necessity for a novel tool that can harness the unique advantages presented by ultra-high field imaging. Such a tool would be well poised to uncover the subtle volume changes—typically around 1%—that are integral to exercise-related studies. The results evidently showed the robustness of DL with respect to ultra-high field MRIs across scanning acquisition protocols.

The performance of deep learning models is largely influenced by the training datasets (Zhang et al., 2020; Guan et al., 2021; Lyu et al., 2022). In order to achieve optimal performance, it is crucial for testing images to follow a similar distribution to that of the training images. To increase the generalizability of our DL, we included MRIs from different populations, scanners, and acquisition protocols by mixing multiple datasets as a bigger training set, following the methodology of previous literature (Balboni et al., 2022). In Supplementary Figure S4, we presented sample images from each dataset. This graphical representation qualitatively underscored the observable domain gaps between the training and evaluation datasets. To offer a more intuitive insight, we employed Supplementary Figure S5 to visualize the latent distribution of all datasets using t-SNE (van der Maaten and Hinton, 2008) dimension reduction. Notably, our analysis revealed that the training datasets and evaluation datasets exhibited minimal overlap within the latent space. This observation underscored the inherent diversity in both our training and evaluation data, corroborating the effectiveness of our approach. However, we found that increasing generalizability may lead to a slight decrease in performance on specific datasets. To address this issue, we selected the PMC atlas set as the training set based on visual inspection and tested on TOMCAT. Our results demonstrated that DL ‘s performance was further improved, with the median VPD decreasing from 1.23 to 0.29% for the left hippocampus and from 0.88 to 0.24% for the right hippocampus (Supplementary Figure S6). This suggests that a specifically developed deep learning model is preferable over a generic toolbox like FSL and Freesurfer. The nnU-Net model involved in the DL of this study provides an accessible solution for neuroscientists to develop their own deep learning models without requiring too much expert knowledge. All needed is to prepare a training dataset visually similar to the clinically-concerned target data, and train the model for a few hours with graph processing units (GPU). Another potential solution is to build a multi-atlas based deep learning model, which automatically selects a suitable set of atlases with respect to histograms or deep image features and performs segmentation with its corresponding model.

In the BLME model, the normalized time from baseline by month is the only variable. This may affect its statistical power on the ADNI3 dataset since it cannot track disease-related changes. In our future work, a more sophisticated model and more data such as the ADNI1 dataset will be included to detect disease-related changes and statistically compare the performance of various pipelines in distinguishing between diagnostic groups.

Only 3T atlases were adopted as the training dataset in this study, which may have potentially affected the performance of the deep learning model when applied to 7T data. However, our findings indicate that DL was not affected by the field strength and voxel dimension, even without any preprocessing or inhomogeneity correction. It is surprising that DL was also able to overcome the challenge of high intensity inhomogeneity in 7T images (Supplementary Figure S7), possibly due to its extensive data augmentation, which effectively varies the intensity profiles and regularizes the segmentation model. Nevertheless, the limited training data used in this study remains a key limitation, and future studies will aim to incorporate submillimeter ultra-high field MRIs with expert hippocampal annotation into the training dataset to further enhance the robustness and performance of the evaluated deep learning model.

The relatively small sample size of TOMCAT (*N* = 7) also limits the impact of our study. Although we observed non-significant improvements over Freesurfer and FSL for both hemispheres due to their high variability in VPD and ICC, we included a submillimeter 3T dataset (*N* = 45) to strengthen our findings. Additionally, we included a larger 7T dataset (*N* = 34) and the well-established ADNI3 dataset (*N* = 64) but were unable to conduct test–retest statistics in terms of ICC and VPD due to the various timepoints and intervals. Future studies will involve more test–retest data from 7T scanners to improve the generalizability of our findings. Overall, as DL consistently outperformed other methods on multiple datasets, we conclude that deep learning is a reliable and promising approach for longitudinal studies of exercise in healthy adults.

## 5 Conclusion

In conclusion, using VPE and CC, we demonstrate that deep learning has remarkable accuracy and precision and outperforms conventional methods. We also show that DL has superior test–retest reliability, as assessed by VPD and ICC, which is within the level of age-related atrophy. Additionally, we apply the BLME model to a large longitudinal dataset with ultra-high field MRIs, showing that deep learning allows to sensitively detect subtle changes over time and the slight differences across subjects. Our results not only indicate that deep learning predicts a more reliable hippocampal volume than conventional approaches, especially on ultra-high field MRIs, it is also more reliable in its performance which is promising for longitudinal studies involving hippocampal measures.

## Data availability statement

The original contributions presented in the study are included in the article/Supplementary material, further inquiries can be directed to the corresponding authors.

## Ethics statement

The studies involving humans were approved by Research Ethics and Integrity, The University of Queensland. The studies were conducted in accordance with the local legislation and institutional requirements. Written informed consent for participation was not required from the participants or the participants’ legal guardians/next of kin in accordance with the national legislation and institutional requirements.

## Author contributions

JL wrote the first draft of the manuscript and performed the statistical analysis. JL, FN, and XT contributed to conception and design of the study. FN and XT revised the manuscript and helped to embellish language. PB provided the expert consultations and suggestions. All authors contributed to manuscript revision, read, and approved the submitted version.

## Footnotes

^1^ https://surfer.nmr.mg.harvard.edu

^2^ https://www.fil.ion.ucl.ac.uk/spm/
