# Supplementary material for: Toward hippocampal volume measures on ultra-high field magnetic resonance imaging: a comprehensive comparison study between deep learning and conventional approaches
Source: Front Neurosci. 2023 Dec 14;17:1238646. doi: 10.3389/fnins.2023.1238646 (PMC10752989; doi:10.3389/fnins.2023.1238646)
Supplement: Supplementary file 2 [file Data_Sheet_2.docx]

Supplementary Material

Towards hippocampal volume measures on ultra-high field magnetic resonance imaging: A comprehensive comparison study between deep learning and conventional approaches

Junyan Lyu, Perry Bartlett, Fatima Nasrallah^*^, Xiaoying Tang^*^

*** Correspondence:**Dr. Fatima Nasrallah
[f.nasrallah@uq.edu.au](mailto:f.nasrallah@uq.edu.au)

Dr. Xiaoying Tang

[tangxy@sustech.edu.cn](mailto:tangxy@sustech.edu.cn)

# Statistical Methods

Regarding volume percentage error (VPE) on ADNI-HarP, we conducted Wilcoxon matched-pairs signed rank tests, as the Kolmogorov-Smirnov normality tests indicated that DL did not adhere to normal distribution for both the left and right hippocampus.

For left hippocampal volume percentage differences (VPEs) on HCP, all methods did not pass the normality tests, thus non-parametric tests were employed.

In the context of the right hippocampus, when comparing DL with Freesurfer and FSL, non-parametric tests were used. However, in the comparison of DL with DARTEL, where only Freesurfer and FSL exhibited non-normality for right hippocampal volumes, paired t-tests (parametric tests) were employed.

For both the left and right VPEs on TOMCAT, paired t-tests (parametric tests) were carried out, as normality tests were not passed for all methods involved.

# Supplementary Figures


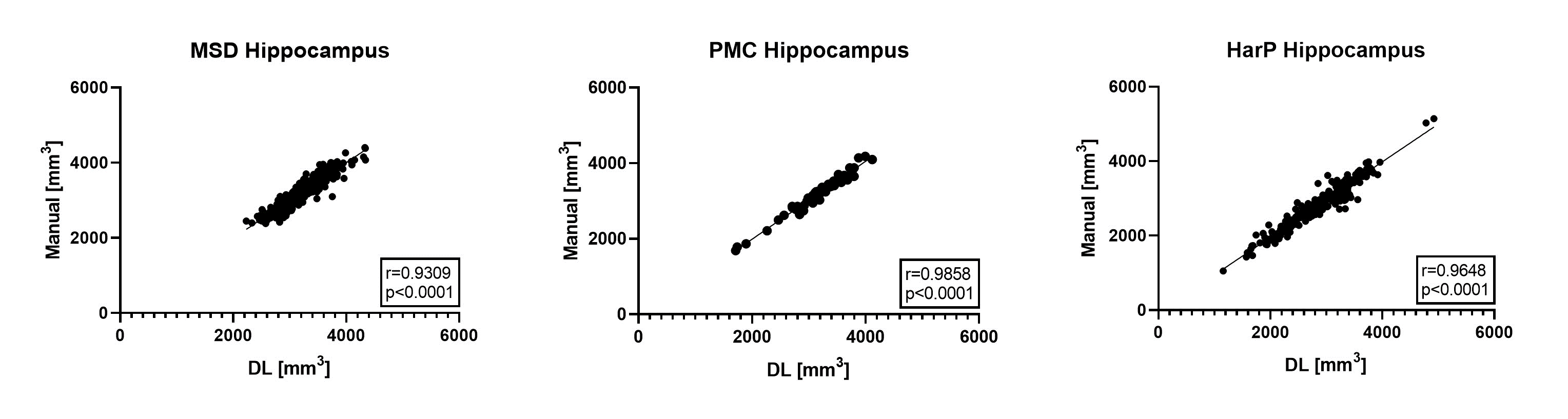


**Supplementary Figure 1.** Pearson correlation coefficient analyses between DL and manual derived volumes of the hippocampus on MSD, PMC and HarP: scatter plots wherein each solid line shows a linear fit. A higher r value indicates stronger correlation.


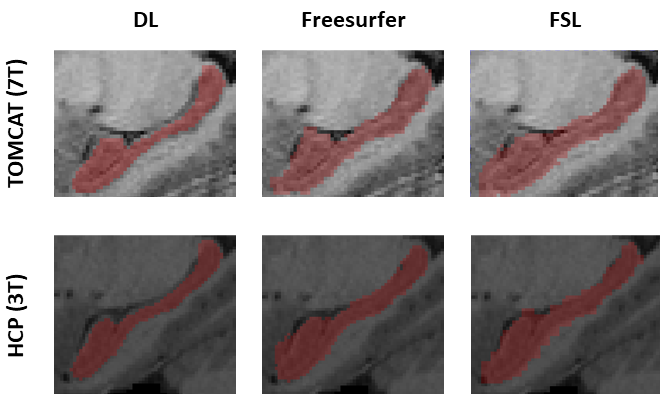


**Supplementary Figure 2.** Qualitative comparisons of DL, Freesurfer and FSL on two submillimeter MRI datasets including TOMCAT (7T) and HCP (3T). The examples were randomly selected from the datasets.


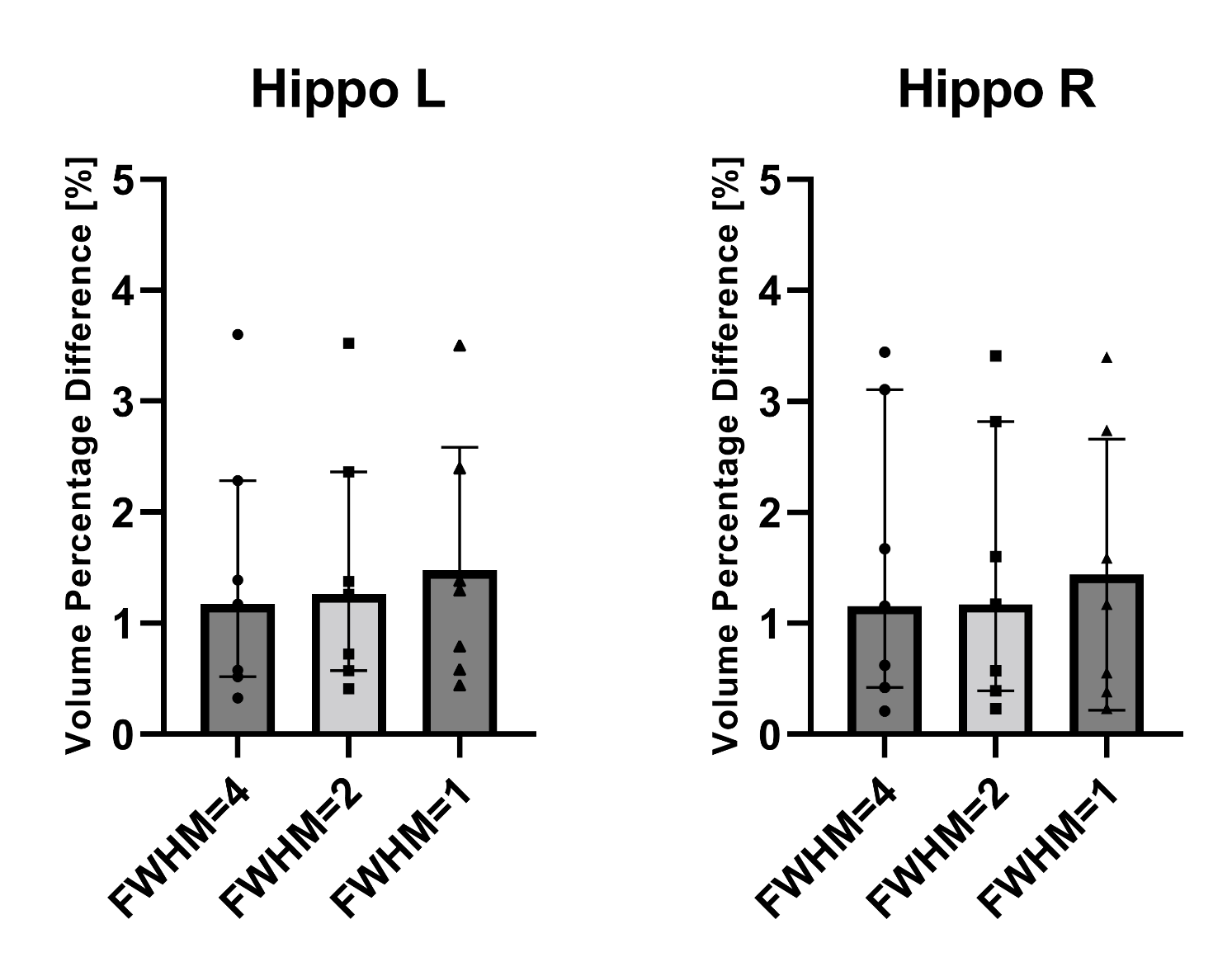


**Supplementary Figure 3.** Boxplots with medians and interquartile ranges of volume percentage differences on TOMCAT for different full-width at half maximum (FWHM) of the Gaussian smoothing kernel in DARTEL.


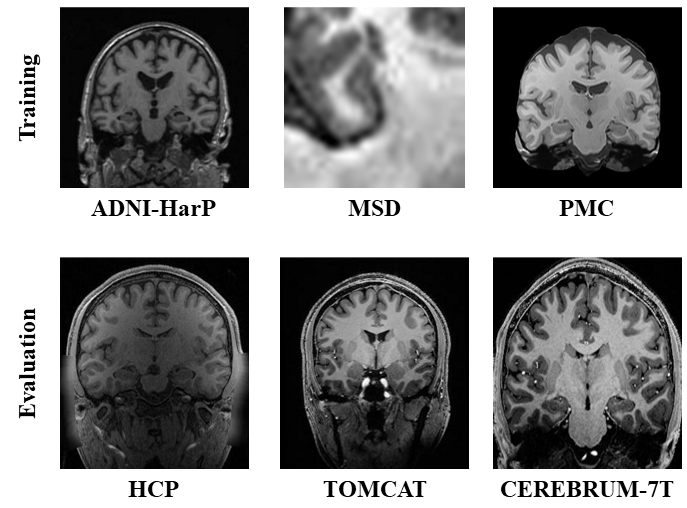


**Supplementary Figure 4.** Qualitative comparisons of MRIs from different datasets. The upper row displays images from the training datasets, while the lower row showcases images from the evaluation datasets.


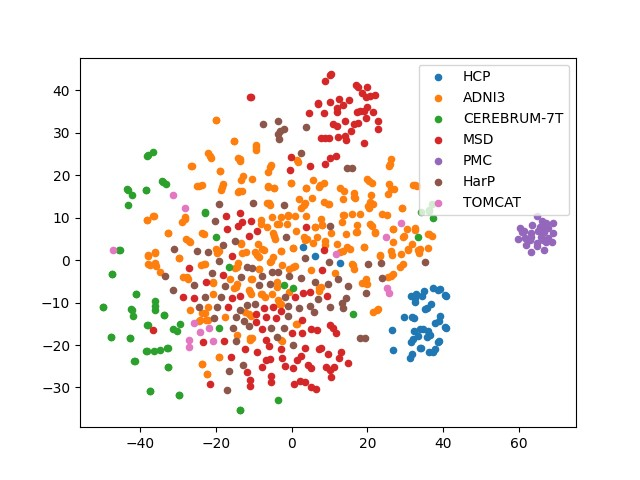


**Supplementary Figure 5.** t-SNE visualization of the latent distribution of training datasets (ADNI-HarP, MSD, and PMC) and evaluation datasets (HCP, TOMCAT, and CEREBRUM-7T). The depiction of the spatial arrangement in the latent space illustrates the domain gap between the training datasets and the evaluation datasets.


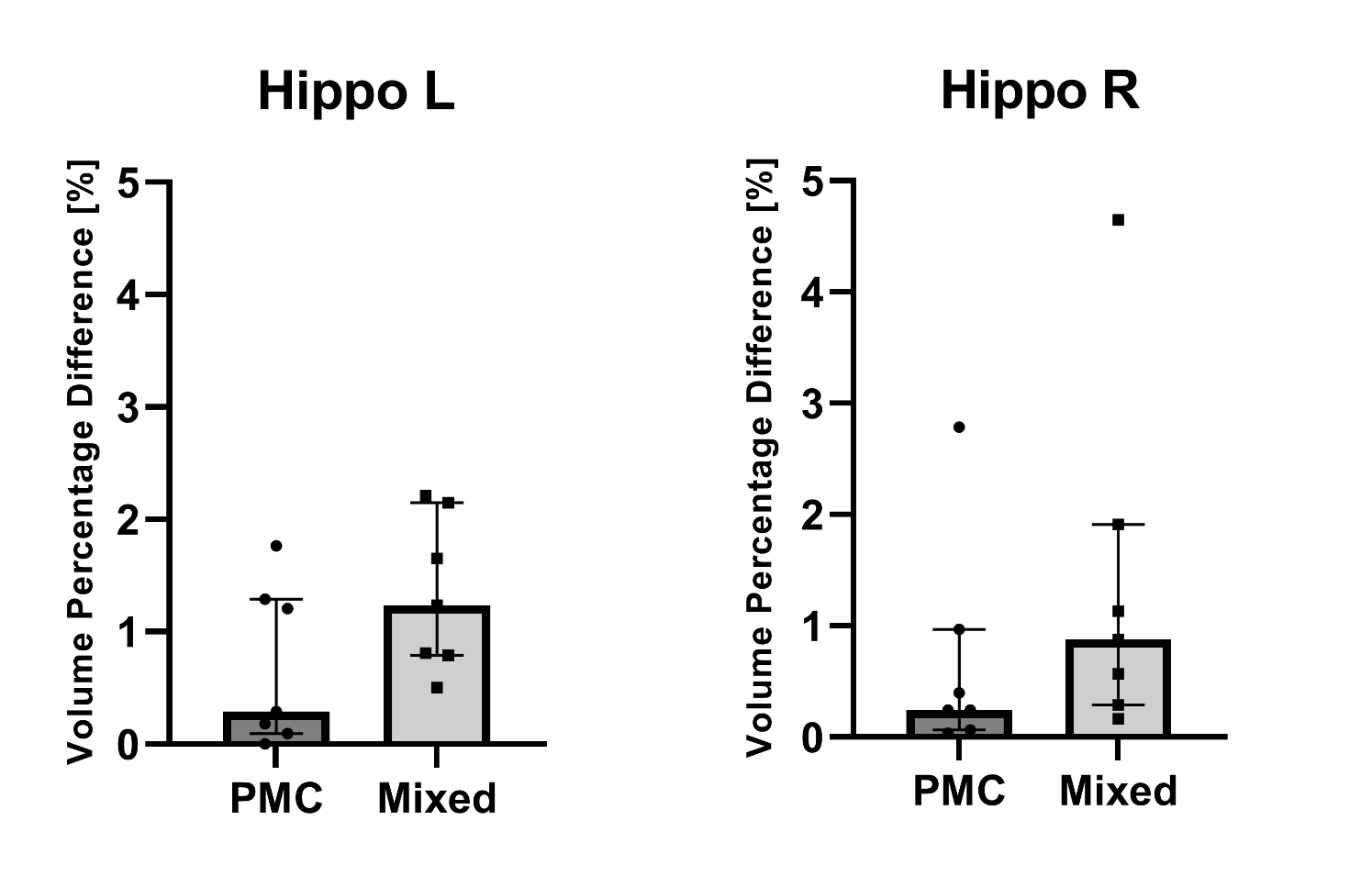


**Supplementary Figure 6.** Boxplots with medians and interquartile ranges of volume percentage differences for different training datasets on TOMCAT. Mixed denotes mixing all the three datasets as a single training dataset.


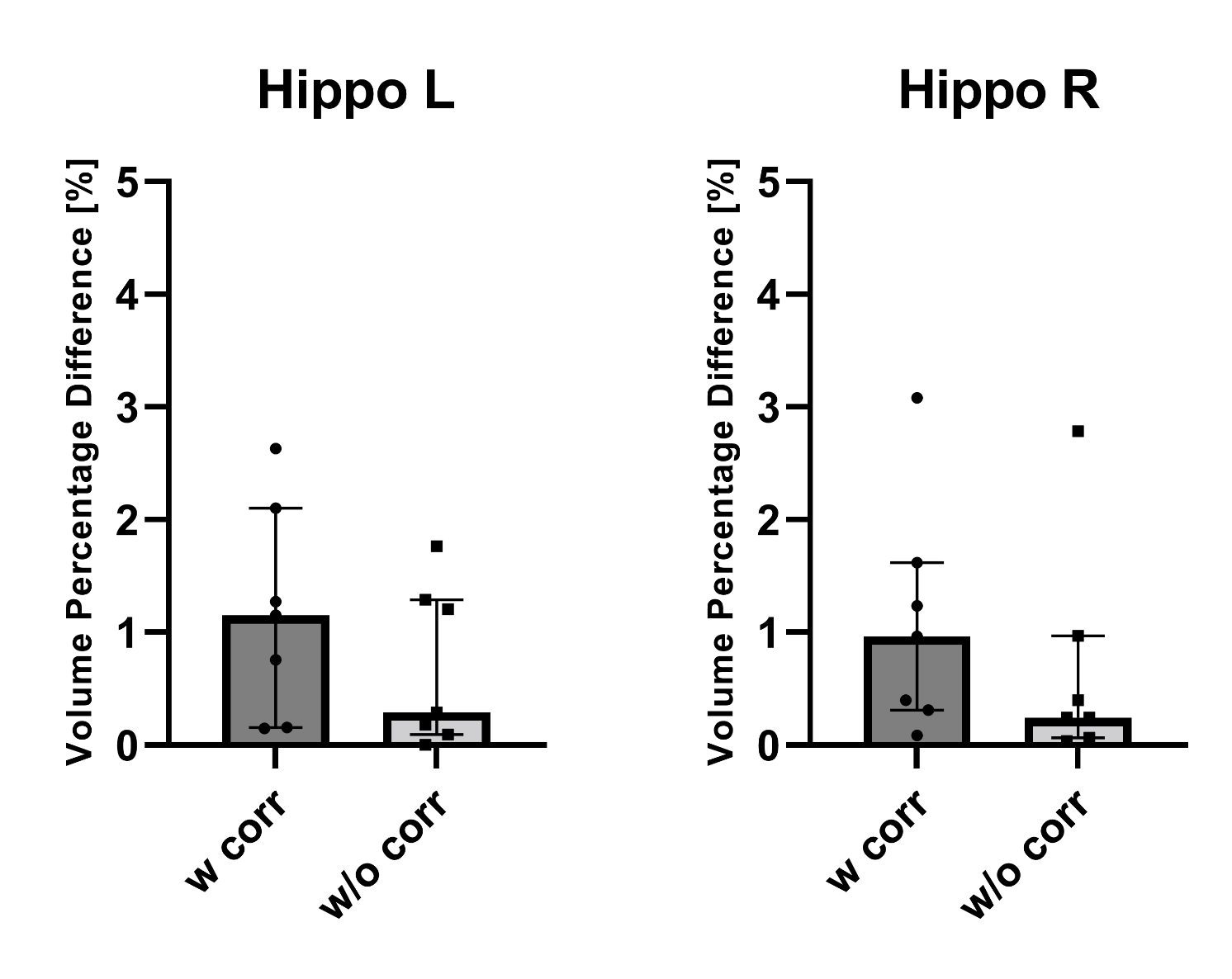
**Supplementary Figure 7.** Boxplots with medians and interquartile ranges of volume percentage differences for different regularization methods used in DL on TOMCAT. “w corr” denotes using N4 bias field correction to preprocess the MRI. “w/o corr” means without bias field correction.


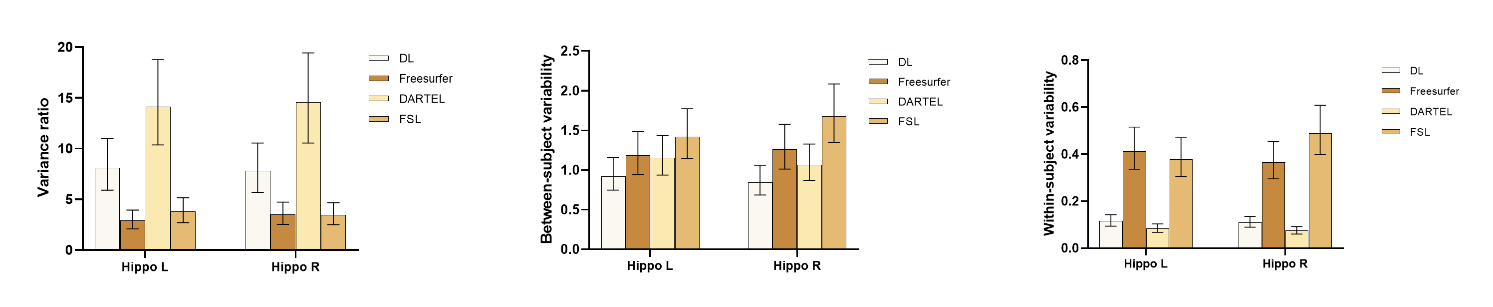


**Supplementary Figure 8.** Boxplots with means and 95% confidence intervals of the variance ratio, between-subject variability, and within-subject variability for all four methods under comparison on HCP. The higher variance ratios indicate better discrimination between subjects, and higher within-subject reproducibility between the test-retest conditions.

# Supplementary Tables

|  | Median | Mean | 25^th^ Percentile | 75^th^ Percentile | Standard Deviation |
| --- | --- | --- | --- | --- | --- |
| DL | 0.29% | -0.11% | -3.11% | 3.31% | 4.96% |

**Supplementary Table 1.** Statistical values including median, mean, 25^th^ percentile, 75^th^ percentile and standard deviation of volume percentage error (VPE) for each method for hippocampal volume measurements on MSD. A VPE closer to zero indicates a lower systematic error, and a lower standard deviation suggests a higher precision. We couldn't provide left or right hippocampal statistics on MSD due to the absence of orientation information in the cropped images.

|  | Median | | Mean | | 25^th^ Percentile | | 75^th^ Percentile | | Standard Deviation | |
| --- | --- | --- | --- | --- | --- | --- | --- | --- | --- | --- |
|  | L | R | L | R | L | R | L | R | L | R |
| DL | -0.96% | -0.33% | -1.33% | -0.75% | -3.08% | -2.18% | 0.88% | 1.15% | 2.95% | 2.59% |

**Supplementary Table 2.** Statistical values including median, mean, 25^th^ percentile, 75^th^ percentile and standard deviation of volume percentage error (VPE) for each method for hippocampal volume measurements on PMC. A VPE closer to zero indicates a lower systematic error, and a lower standard deviation suggests a higher precision.
